# Does Dietary Intake by Tehranian Adults Align with the 2005 Dietary Guidelines for Americans? Observations from the Tehran Lipid and Glucose Study

**DOI:** 10.3329/jhpn.v29i1.7564

**Published:** 2011-02

**Authors:** Parvin Mirmiran, Firoozeh Hosseini-Esfahani, Mahsa Jessri, L. Kathleen Mahan, Niloofar Shiva, Fereidoun Azizi

**Affiliations:** ^1^ Obesity Research Center, Research Institute for Endocrine Sciences, Shahid Beheshti University of Medical Sciences, Tehran, Iran; ^2^ Faculty of Nutrition Sciences and Food Technology, National Nutrition and Food Technology Research Institute, Shahid Beheshti University of Medical Sciences, Tehran, Iran; ^3^ Department of Pediatrics, School of Medicine, University of Washington, Seattle, WA, USA; ^4^ Research Institute for Endocrine Sciences, Shahid Beheshti University of Medical Sciences, Tehran, Iran; ^5^ Endocrine Research Center, Research Institute for Endocrine Sciences, Shahid Beheshti University of Medical Sciences, Tehran, Iran

**Keywords:** Adherence, Adults, Calorie intake, Diet, Guidelines, Nutrition, Iran

## Abstract

The aim of this study was to compare dietary intakes by Tehranian adults with recent dietary guidelines for the Americans. The study made a cross-sectional assessment of the dietary patterns of Tehranian adults using a validated food-frequency questionnaire. It included 2,510 subjects (1,121 men and 1,389 women) aged 19-70 years. They were the participants of the third follow-up survey of the Tehran Lipid and Glucose Study (2005-2008). The dietary patterns were assessed using the latest World Health Organization (WHO)'s nutritional goals and Dietary Guidelines for the Americans Adherence Index (DGAI) 2005. The mean [standard deviation (SD)] DGAI score for this population was 8.31 (1.9). Participants in the highest quartile category of DGAI were more likely to be female, older, non-smoking, and physically active than those in the lowest quartile category (p<0.001). Percentage of participants meeting the DGA recommendations waslow, especially for starchy vegetables (2.3%), orange vegetables (16.2%), lean meat (9.2%), grains (12.0%), and legumes (6.4%). Over-consumption of grains was observed in almost half of the participants while approximately 20% of the subjects over-consumed milk and meat groups. Intakes of most nutrients examined were significantly associated with the DGAI 2005 score (p<0.001), except for vitamin E, vitamin B12, and vitamin D. The least adherence with the WHO goals was observed with n-3 PUFAs, sodium, fruit, and vegetable intakes. The results revealed that the dietary patterns of most Tehranian adults did not comply with the 2005 DGA and nutritional goals of WHO/Food and Agriculture Organization.

## INTRODUCTION

The importance of maintaining a healthful diet in preventing diet-related diseases has been emphasized (1, 2). The Food and Agriculture Organization (FAO), in collaboration with the World Health Organization (WHO), has developed a series of international dietary recommendations to prevent chronic diseases and promote good health (3). Seve-ral countries also have dietary recommendations (4–7), and those from the USA have been further updated (8).

The Dietary Guidelines for the Americans Adherence Index (DGAI) 2005 is aimed at assessing the adherence of adult populations to these guidelines (9) and, compared to previous tools, it is a preferable measure of the quality of diet as it penalizes over-consumption of energy-dense foods—a shortcoming of earlier indices (10). It also assesses discretionary calorie intakes from solid fats and added sugars and as well assesses separately the five groups of vegetables (9, 11). The national nutritional guidelines in Iran cover mainly the general qualitative dietary recommendations for healthful eating (12) and, hence, studies conducted on dietary habits of Iranians have used earlier versions of the food guide of the United States Department of Agriculture (USDA), showing that Iranian adults are transitioning to diets high in fat and sweets (13, 14) and low in meats and dairy products (15). However, updates in the DGA 2005 are more precise and informative compared to earlier versions, emphasizing the necessity of re-assessing the dietary patterns. The present study evaluated the dietary compliance of Tehranian adults with the latest WHO/FAO nutrition goals and key recommendations of the USDA food guide, using the DGAI 2005.

## MATERIALS AND METHODS

### Study population

The Tehran Lipid and Glucose Study (TLGS) is a community-based prospective study, performed on a sample of residents referring to three health centres in district no.13 of Tehran, the capital of Iran (16). This study, originally designed to prevent non-communicable disorders (NCDs) using a programme to promote healthy lifestyles and reduce the risk factors of NCDs (17), began in 1999, and baseline measurements are being followed every three years. Of 9,602 individuals aged 19-70 years, the participants of the Third TLGS Follow-up Survey (2005-2008), 2,881 (30%) adults were randomly selected for dietary assessment. Men and women were proportionately distributed across five 10-year age-groups to enable generalization of results to all ages and both sexes (18). Excluded were subjects with missing data on any of the following: age, gender, physical activity, and any anthropometrical measurement (n=97). Dividing the reported energy intake (rEI) by predicted energy requirement (pER), the same as estimated energy requirement (EER) resulted in ratios which, if they did not qualify for the ±2 standard deviation (SD) range, were considered inaccurate reports of dietary energy intake (under- and over-reporting) (19). These (n=274) subjects were also excluded from the study. Finally, data for 2,510 subjects—1,121 men and 1,389 women—were analyzed.

### Energy requirement

The EERs of the participants were calculated, using the dietary reference intake (DRI) based on age, gender, weight, height, and physical activity levels (20). A physical activity questionnaire was used for assessing various aspects of physical activity and energy expenditure (21). Each activity category was presented to subjects as a list of examples of common activities of daily life. The participants were asked to specify time spent for sleeping and also the frequency and time spent on activities of light, moderate, hard and very hard intensity during the previous year. A metabolic equivalent (MET) value, the metabolic rate during physical activities of varying intensities as multiples of resting metabolic rate (kcal/kg/hour), was calculated to interpret the daily physical activity of each participant for the estimation of energy requirements (22, 23).

### Other measures

Weight was measured with subjects minimally clothed, standing on digital scales (Seca, Germany) without shoes, and was recorded to the nearest 100 g. For height, subjects were measured in standing position without shoes, using a stadiometer (17). Body mass index (BMI) was calculated. Waist-circumference (WC) was measured at the umbilicus level using a non-stretch tape. Trained physicians collected additional information on age, smoking behaviour (according to the WHO guidelines), medical history, and current use of medications during face-to-face interviews (17, 24).

Blood glucose and blood pressure were measured by methods described earlier (17). Diabetes was diagnosed based on the latest standard protocols of the American Diabetes Association (ADA) (25) which considers fasting plasma glucose ≥126 mg/dL, two-hour plasma glucose ≥200 mg/dL, or drug treatment for hyperglycaemia. Hypertension was diagnosed according to the criteria of the Joint National Committee (JNC-VII) at systolic blood pressure of ≥140 mmHg, diastolic blood pressure of ≥90 mmHg, or drug treatment for a previous diagnosis of hypertension (26).

### Dietary assessment

Trained dietitians collected dietary data using a 168-item semi-quantitative food-frequency questionnaire (FFQ) (27, 28). Portion sizes for each FFQ food item were specified according to the USDA portion sizes and, in some cases, household measures. The participants reported the frequency of their consumption for each food item during the previous year on a daily, weekly, or monthly basis. Since the Iranian food-composition table (FCT) (29) is incomplete and provides data only on a few nutrients to analyze food and beverages for energy and nutrients, we used FCT of the USDA (30). For the estimation of trans-fat contents of foods, not included in FCT of the USDA, McCance and Widdowson's composition of foods was used (31). However, for some Iranian food items (e.g. dairy products such as *kashk*), not in FCT of the USDA, we used the Iranian FCT. For mixed items (e.g. pizza), nutrients were calculated based on reported basic ingredients and usual restaurant recipes; grammes of consumed food items were converted to cup- and ounce-equivalents using the ‘food link pyramid database series’ (32).

#### Nutrient intake goals of WHO/FAO

We evaluated the compliance of dietary patterns with the WHO/FAO nutrition goals. The main dietary components of the WHO/FAO goals include total fat, polyunsaturated fatty acids (PUFA), monounsaturated fatty acids (MUFA), saturated fatty acids (SFA), trans-fats, protein, carbohydrate, free sugars, n-3 and n-6 fatty acids, sodium, fruits and vegetables, total fibre, and non-starch polysaccharides (3).

#### Dietary Guidelines for Americans Adherence Index

This index contains calorie-specific guidelines for 10 different energy intakes based on the energy and nutrient requirements of individuals and assesses adherence to 16 key recommendations of the DGA 2005. The original DGAI has a maximum score of 20 points, 11 of which assess the ‘food intake recommendations’ and nine assess ‘healthy choice recommendations’ (8, 9). In our study, only 19 DGAI scores were attainable, and a subscore of healthy choice recommendations (which considers alcohol consumption) was not calculated for our population, since Iranians, based on their religious beliefs, do not drink alcoholic beverages. The maximum value for items of this index is 1.0 (consumer) and, for most items, there is a partial credit of 0.5 for persons not meeting recommendations fully but consuming over 33% of the recommended amount (intermediate consumer). Zero point referred to those consuming <33% of the recommended amount (non-consumer). This index also penalizes for over-consumption of energy-dense foods (i.e. meat, dairy, grains, and starchy vegetable groups) to limit the likelihood of the maximum score being obtained solely by over-consumption of energy (over-consumer) (9). An example of the DGAI 2005 used in the present study is presented for 2000 kcal in the Appendix.

*Food intake subscore:* Each of the five vegetable subgroups (orange, dark green, starchy, other vegetables, and legumes) was scored separately on a weekly basis (8) (Appendix). The consumption patterns of fruits, grain, milk, meat, and various fruits and vegetables were evaluated based on the daily energy intake by the participants. Considering the recommendations of the DGAI 2005, legumes were assigned to the meat group but only for those who did not consume the meat group enough and had not met the 1.0-point criterion for this group; the intake of extra legumes was counted towards the vegetable group (8).

Discretionary energy is energy derived from solid fat and added sugar. Since the DGAI 2005 considers the solid fat component of discretionary energy in the ‘saturated fat’ item of the ‘healthy choice recommendations’, the percentage of energy from added sugar is only calculated in the ‘food intake subscore’ (8). Mixed food items, e.g. cookies, snacks, confectioneries, etc., which did not exactly belong to one specific food group, were considered a share of discretionary energy, and their intakes contributed to scoring ‘healthy choice recommendations’ (32).

#### Healthy choice recommendations

These recommendations, not dependent on estimated energy need, are the same for all energy levels and assess the percentage of whole grain, fibre intake, and sodium intake; five of these are related to fat and cholesterol intake, including low-fat milk and meat choices (Appendix).

### Statistical analysis

All statistical analyses were performed using the SPSS software (PC version 16.0). The DGAI score was normally distributed and was divided into quartile categories. Chi-square test was performed to determine the percentage of females, low-activity participants, smokers, and subjects diagnosed with diabetes and hypertension, in two age-groups. To compare the characteristics of the participants and nutrient intakes across quartiles of the DGAI 2005, analyses of covariance were used with adjustment for age and/or gender and energy intake.

Given that the DGAI score is a continuous measure, analysis of p-trend was used for checking whether the characteristics of subjects and their nutrient intakes were related in a monotonically-increasing or decreasing manner with the DGAI score. The linear regression coefficient was used for continuous dependent variables, and logistic regression coefficient was used for dichotomous dependent variables. The mean food group intakes, discretionary calories, and energy percentages as discretionary energy were analyzed by gender and two age-categories and adjusted for reported energy intake, using analysis of covariance. The percentage of consumers, non-, intermediate- and over-consumers in each component of the DGAI score was analyzed for each gender separately. Chi-square test was used for testing the differences between genders among compliers with the WHO/FAO nutritional goals. Logistic regression analysis was used for determining the risk of diabetes and hypertension across the quartile categories of discretionary calorie intakes.

### Ethical approval

Informed written consent was obtained from all the subjects. The Research Council of the Research Institute for Endocrine Sciences, Shahid Beheshti University of Medical Sciences, approved the study proposal.

## RESULTS

The mean (SD) age of the participants was 39.5 (13.4) years: 73.7% of men and 79.4 % of women were aged 19-50 years. The mean (SD) DGAI score was 8.31 (1.9) (range 2.5-15.0), a score shown to be associated with several characteristics of the participants (Table 1). The percentage of the female participants increased significantly in higher quartiles of the DGAI score (29.2% vs 25.9%; p=0.04) while having a decreasing trend in males (17.4% vs 30.3%; p<0.001). Participants in the highest quartile category of the DGAI score were more likely to be older (49.1 years vs 35.3 years—males; 41.1 years vs 34.7 years—females; p<0.001) than those in the lowest quartile category.

**Table 1.** Characteristics of participants by quartile categories of the DGAI 2005: the Tehran Lipid and Glucose Study*

Characteristics	DGAI 2005 quartile category									
	Male					Female				
	1	2	3	4	p trend†	1	2	3	4	p trend†
DGAI range‡	2.50-7.00	7.25-8.25	8.50-9.50	9.75-15.00		2.75-7.00	7.25-8.25	8.50-9.50	9.75-15.00	
No. of participants (%)	340(30.3)	305(27.2)	281(25.1)	195(17.4)	<0.001	360(25.9)	324(23.4)	299(21.5)	406(29.2)	0.04
DGAI score, median	6.25	7.75	9.00	10.50		6.25	7.75	9.00	10.75	
Healthy choice subscore, median¶	2.50	3.75	4.00	4.75		2.00	3.25	3.75	4.75	
Food intake subscore, median§	3.50	4.00	5.00	6.00		4.00	4.50	5.50	6.50	
Age (years)	35.3	38.9	42.7	49.1	<0.001	34.7	36.4	39.1	41.1	<0.001
BMI (kg/m2)	26.30	26.20	27.20	26.80	0.13	27.10	27.30	27.00	26.90	0.41
Waist-circumference (cm)	93.0	93.3	95.7	95.6	0.57	85.2	85.0	82.32	82.1	0.03
Participants of light activities (%)**										
19-50 years	53.1	47.4	45.9	42.9	<0.001	42.3	44.0	40.2	37.5	<0.001
50-70 years	54.1	40.7	45.2	43.8	0.08	52.1	42.9	46.8	47.2	0.36
Current smokers (%)**	26.9	23.7	18.5	15.3	<0.001	4.7	4.3	3.5	1.8	<0.001
Diagnosis of hypertension (%)**	42.8	40.1	39.6	35.4	<0.001	41.0	35.9	33.5	31.6	<0.001
Diagnosis of diabetes (%)**	12.4	7.3	6.5	5.2	<0.001	9.4	7.1	6.7	6.9	0.05

*Values are reported as mean or percentages and are adjusted for age, except for age and percentage of participants of light activities;

†p value for trend was determined using the linear regression coefficient for the DGAI score for continuous variables (age, BMI, and waist-circumference) and for the dichotomous variables (current smoker, hypertension, diagnosis of diabetes, and physical activity), logistic regression coefficient for the DGAI score was administered;

‡Possible point range from 0 to 19;

¶Possible points range from 0 to 8 and was assessed at the same level for all subjects;

§Possible points range from 0 to 11 and was assessed at 10 different energy levels;

**Physical activity values were categorized according to the intensity using the ACSM/CDC guidelines. Smoking status was classified according to the WHO guidelines. Hypertension was defined according to the JNC-VII (Joint National Committee) criteria. Diabetes was diagnosed based on the latest standard protocols of the American Diabetic Association. ACSM/CDC=American College of Sports Medicine/Centers for Disease Control and Prevention; BMI: Body mass index; DGAI=Dietary Guidelines for the Americans Adherence Index; WHO=World Health Organization

Table 2 shows the mean intakes of food group by age and sex. After adjusting for energy intake, women in both age-categories consumed significantly more fruits, total vegetables (dark-green vegetables, orange vegetables, and other vegetables), and milk compared to their male peers (p<0.001). Older subjects (aged 50-70 years) in both sexes consumed more fruits, total vegetables (dark-green vegetables, orange vegetables, other vegetables), and whole grains (p<0.001). However, their intakes from total grain, other grains, lean meat, milk, and discretionary calories were significantly lower (p<0.001). Women aged 19-50 years consumed significantly more discretionary calories as a share of total energy compared to their male counterparts (32.1 vs 29.8%, p<0.001). About 30% of daily calories in our population were provided by discretionary energy (solid fat and added sugar) (Table 2). Participants in the higher quartile of discretionary calorie intake had higher probability of having hypertension [odds ratio (OR)=1, 1.33, 1.96, 2.16; p<0.001] and diabetes [OR=1, 2.01, 2.62, 3.00; p=0.04] compared to subjects in the first quartile, after adjustments for age and gender (data are not shown).

**Table 2.** Number of cup/ounce equivalents and discretionary calories consumed from each food group of the Dietary Guidelines for the Americans 2005 by sex and age in adult participants of the Tehran Lipid and Glucose Study

Intake of food groups	Male		Female	
	19-50 years (n=826)	51-70 years (n=295)	19-50 years (n=1, 103)	51-70 years (n=286)
Fruit (cup-equi/day)				
Unadjusted	2.10 (1.77)*	2.52 (2.1)	2.06(1.60)	2.37(1.86)
Adjusted†	1.86‡, ¶ (0.05)	2.28‡ (0.10)	2.24¶ (0.04)	2.62 (0.10)
Vegetables (cup-equi/day)				
Unadjusted				
Adjusted†	2.27 (1.33)	2.72 (1.38)	2.61 (1.72)	2.93 (1.70)
Dark-green vegetables (cup-equi/week)	2.05‡, ¶ (0.05)	2.57‡ (0.08)	2.78¶ (0.04)	3.09 (0.08)
Unadjusted	4.5 (4.1)	6.02 (5.18)	5.27 (5.17)	6.57 (5.23)
Adjusted†	4.05‡, ¶ (0.16)	5.77‡ (0.30)	5.60¶ (0.14)	8.30 (0.30)
Orange vegetables (cup-equi/week)				
Unadjusted	0.93 (1.71)	1.26 (1.68)	1.40 (2.36)	1.51 (2.22)
Adjusted†	0.82‡, ¶ (0.07)	1.16‡ (0.11)	1.49¶ (0.06)	1.61 (0.11)
Legumes (cup-equi/week)				
Unadjusted	1.20 (1.65)	1.09 (1.38)	1.08 (1.37)	0.87 (0.81)
Adjusted†	1.10 (0.05)	1.03 (0.06)	1.16 (0.04)	0.93 (0.06)
Starchy vegetables (cup-equi/week)				
Unadjusted	1.51 (1.51)	1.61 (1.40)	1.31 (1.23)	1.25 (1.45)
Adjusted†	1.38 (0.04)	1.50 (0.08)	1.40 (0.04)	1.36 (0.08)
Other vegetables (cup-equi/week)				
Unadjusted	5.60 (3.80)	6.53 (3.80)	6.64 (6.01)	0.44 (4.88)
Adjusted†	5.04‡, ¶ (0.15)	6.15‡ (0.24)	7.06¶ (0.13)	8.17 (0.24)
Grains (oz-equi)				
Unadjusted	14.16 (90.57)	9.42 (4.79)	7.68 (3.73)	7.34 (3.93)
Adjusted†	13.53‡, ¶ (2.1)	8.77‡ (0.21)	8.16¶ (1.8)	8.01 (0.21)
Whole grains (oz-equi)				
Unadjusted	3.36 (3.50)	3.80 (4.16)	2.37 (2.69)	2.88 (3.13)
Adjusted†	3.09‡, ¶ (0.10)	3.50‡ (0.20)	2.58¶ (0.09)	3.19 (0.21)
Other grains (oz-equi)				
Unadjusted	10.44 (90.54)	5.26(3.47)	5.58(3.05)	4.82(3.07)
Adjusted†	10.44‡, ¶ (2.12)	5.26‡ (0.17)	5.58¶ (1.82)	4.82 (0.17)
Lean meat and beans (oz-equi)				
Unadjusted	3.72 (2.48)	3.30 (2.36)	2.95 (1.86)	2.55 (1.65)
Adjusted†	3.36¶ (0.06)	3.03 (0.01)	3.22¶ (0.05)	2.83 (0.10)
Milk (cup-equi)				
Unadjusted	2.74 (1.60)	2.50 (1.40)	2.57 (1.60)	2.42 (1.22)
Adjusted†	2.50‡, ¶ (0.04)	2.37‡ (0.07)	2.76¶ (0.04)	2.56 (0.07)
Oils§(g)				
Unadjusted	57.18 (22.6)	49.77 (19.79)	52.87 (21.44)	45.89 (17.70)
Adjusted†	55.45‡ (0.30)	54.59‡ (0.60)	51.90 (0.26)	49.63 (0.61)
Discretionary calories (Kcal)				
Unadjusted	778 (384)	620 (280)	697 (353)	540 (280)
Adjusted†	692‡, ¶ (10.2)	585 (14.4)	760¶ (8.8)	576 (14.7)
Discretionary calories (% energy)	29.8‡, ¶ (10.9)	27.4 (10.7)	32.1¶ (12.3)	27.7 (12.7)

*Mean (SD);

†Means are adjusted for total energy intake using analysis of covariance;

‡Significant difference between genders (p≤0.05);

¶Significant difference between age-groups (p≤0.05);

§Described as fat from either a plant source or from fish source;

equi=Equivalent;

SD=Standard deviation

**Fig. 1.**
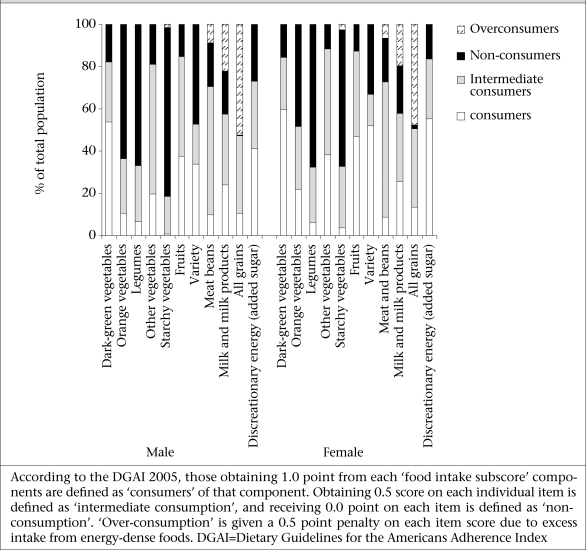
Percentage of male and female subjects within each ‘healthy choice subscore category’ of the Dietary Guidelines for Americans the Adherence Index 2005: the Tehran Lipid and Glucose Study

Figure 1 shows the percentage of participants within the score categories of ‘food intake subscore’ of the DGAI 2005. A very low proportion of the participants met the latest USDA food guide recommendations, especially for starchy vegetables, legumes, lean meat, orange vegetables, and all grain groups. Of the subjects, 52.3% of men and 47.7% of women over-consumed grains, and 22.1% of men and 19.6% of women over-consumed the milk group, thus being categorized as over-consumers; conversely, 20.4% of men and 22.5% of women under-consumed this item.

**Fig. 2.**
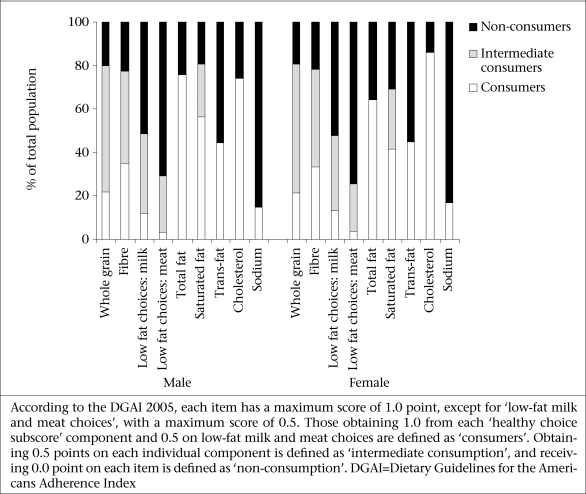
Percentage of male and female subjects within each ‘healthy choice subscore category’ of the Dietary Guidelines for Americans the Adherence Index 2005: the Tehran Lipid and Glucose Study

Figure 2 shows the proportion of males and females within the score categories of ‘healthy choice subscore’ of the DGAI 2005, indicating that <20% of the participants met the recommendations for sodium, low-fat milk, and meat choices. Over two-thirds of the subjects failed to meet the recommendation relating to whole grain.

All nutrients examined were significantly associated with the DGAI score quartiles after adjusting for age, gender, and energy intake, except for vitamin E, B12, and D. Total fat, saturated fat, PUFA, MUFA, n-3 fatty acids, trans-fat, cholesterol, and sodium were inversely associated with the index score (p<0.001). Among macronutrients, there was a positive significant association between the DGAI score and total fibre, carbohydrates, and protein (p<0.001) (Table 3).

**Table 3.** Mean daily intakes of nutrient by quartile categories of the DGAI 2005*, † in adult participants of the Tehran Lipid and Glucose Study

Nutrient	DGAI 2005 quartile category			
	1	2	3	4
DGAI range	2.50-7.00	7.25-8.25	8.50-9.50	9.75-15.00
No. of participants	700	629	580	601
Energy (kcal)	2,219	2,194	2,251	2,374
Total fibre (g)	26.2	28.3	32.0	32.9
Carbohydrate (g)	307	328	336	350
Protein (g)	69.8	71.9	76.8	85.1
Total fat (g)	87.6	77.4	75.4	74.1
Saturated fat (g)	30.8	27.3	25.0	23.0
Polyunsaturated fat (g)	18.8	17.0	16.0	14.3
Monounsaturated fat (g)	31.4	28.0	26.2	23.9
n-3 fatty acids (g)	1.3	1.2	1.1	1.0
Trans-fat (g)	4.8	3.9	3.4	2.4
Cholesterol (mg)	258	223	220	203
Vitamin C (mg)	98	114	147	190
Vitamin E (mg TE)	7.9	7.7	7.8	8.1 NS
Folate (µg)	479	501	530	567
Vitamin B-12 (µg)	4.2	4.0	4.1	4.1 NS
Vitamin D (µg)	1.92	1.92	2.00	2.02 NS
Beta-carotene (µg)	2,253	2,789	3,574	5,469
Sodium (mg)	4,852	4,652	4,360	4,114
Potassium (mg)	2,919	3,168	3,618	4,343
Calcium (mg)	990	1,010	1,092	1,202
Phosphorus (mg)	1,300	1,356	1,450	1,622
Zinc (mg)	10.5	11.2	11.7	12.5
Magnesium (mg)	309	341	374	436
Iron (mg)	13.4	14.2	15.3	16.7
Vitamin A (RAE)	460	467	537	715

*Values are adjusted for total reported energy intake, age, and gender, except for energy intake. The p value for trend was calculated using the linear regression coefficient for the DGAI score for each subject. p<0.001 for all nutrients, except otherwise listed;

†Only micronutrients from food sources are presented.

DGAI=Dietary Guidelines for the Americans Adherence Index;

NS=Non-significant; RAE=Retinol

activity equivalents; TE=Tocopherol

Table 4 shows the percentage of compliers with the WHO/FAO nutrition goals among the male and female subjects. Sex differences in adherence to these nutritional guidelines were significant in most items, with exceptions for sodium, trans fatty acids, and n-6 PUFAs. The greatest compliance with the WHO/FAO nutrition targets was for consumption of free sugars (82.2% in males and 89.8% in females) and cholesterol (73.9% in males and 86.0% in females) while the least adherence was observed with n-3 PUFAs, sodium, and intake of fruits and vegetables in both sexes, with 1.0%, 8.4% and 26.6% for males and 2.4%, 9.1%, and 30.7% for females in each component respectively.

**Table 4.** Percentage of Tehranian population complying with the WHO/FAO nutrition targets in the Tehran Lipid and Glucose Study*

Dietary factor	% of compliers with WHO/FAO nutritional guidelines				
	Recommendation	Male compliers¶		Female compliers	
		No.	%	No.	%
Total fat (%)	15-30	53.2	596	35.7	496
SFA (%)	<10	56.3	631	41.6	578
PUFAs (%)	6-10	42.6	478	49.0	680
n-6 PUFAs (%)	5-8	43.7	490 NS	45.6	634 NS
n-3 PUFAs (%)	1-2	1.0	11	2.4	33
Trans fatty acid (%)	<1	44.3	497 NS	45.0	625 NS
Total carbohydrate (%)	55-75	73.7	826	58.3	810
Free sugars (%)	<10	82.2	921	89.8	1248
Protein (%)	10-15	72.3	810	68.2	947
Cholesterol (mg/d)	<300	73.9	828	86.0	1195
Sodium (g/d)	<2	8.4	94 NS	9.1	126 NS
Fruits and vegetables (g/d)	≥400	26.6	298	30.7	426
Total dietary fibre (g/d)	>25	60.2	675	47.4	659
Non-starch polysaccharides (NSP)	>20	46.1	517	62.2	864

All variables are statistically significant between sexes, except those identified as NS.

*Analyses of significance were performed using Chi-square test;

¶Whole grains, fruits, and vegetables were categorized as NSP;

FAO=Food and Agriculture Organization;

NS=Non-significant.

NSP=Non-starch polysaccharides;

UPFA=Polyunsaturated fatty acid;

SFA=Saturated fatty acid;

WHO=World Health Organization;

## DISCUSSION

The results revealed that the dietary patterns of most Tehranian adults did not comply with the DGAI 2005 as over two-thirds of this population obtained less than 9.5 points (half the possible score) in the DGAI.

This is the first study to assess the dietary adherence of an Iranian adult population to the DGAI 2005 and the latest WHO/FAO nutritional goals. Previous studies conducted on Iranian populations used older versions of the dietary adherence tools (e.g. Healthy Eating Index—HEI) (15), highlighting the need for re-assessments. The mean DGAI score for our population was similar to that of American adults (9), taking into account the fact that the maximum DGAI score in our population was 1.0 point less than the original DGAI, following the elimination of the alcohol intake subscore.

Women, older adults, non-smokers, and active individuals (aged 19-50 years) consumed a diet more consistent with the DGAI 2005, a result similar to that of an American study using the same index (9). However, conversely, our results showed significant associations between the DGAI score and physical activity, hypertension, and diabetes. The study by Kant *et al*. reported a significant relationship between the DGAI and fasting insulin (11), indicating that the HEI, the recommended food score, and dietary diversity score were significantly associated with age, gender, smoking status, and physical activity. These indices were inversely related to BMI, plasma glucose, and haemoglobin AIc. The HEI was not inversely related to blood pressure and serum cholesterol while two other indices showed a significant association (33).

According to the DGAI 2005, 12-20% of total energy could be gained from discretionary calories (8). However, of concern is the finding that Tehranian adults get approximately one-third of their total energy from discretionary calories, and those in the higher quartile of discretionary calorie intake have over two times the risk of having hypertension and diabetes, similar to a previous study (34).

According to the HEI assessed earlier for our population (15), 45.4% of the subjects obtained a ‘good score’ for cereals while the present study showed that only 12.2% of the Tehranian adults were ‘consumers’ and obtained the full score for cereal consumption, and 49.7% were categorized as ‘over-consumers’, explained possibly by the fact that the HEI does not consider an upper-limit for consumption of grains while the DGAI does. The prevalence of grain over-consumption is high in developing countries because grains are staple food and are, thus, over-consumed by most people due to their low cost and easy accessibility (13). Based on previous studies (15), ≤25% of Tehranian adults are milk-consumers, with even less over- and under-consumers, indicating a great disparity in relation to milk consumption. In the lean meat group, the number of under-consumers was higher than the number of over-consumers, showing the need for implementation of appropriate policies, since meat-group items are among the more expensive foods in Iran (13). Nutrition education, with a focus on legumes as appropriate substitutes for the meat group should be given.

Not surprisingly, individuals in the highest quartile category of DGAI were consuming more micronutrients while their intakes of macronutrients were more in alignment with recommendations compared to those in the lower quartile categories of the DGAI, findings in line with those of earlier studies. A high DGAI score was positively associated with healthy lifestyle choices and optimal intakes of micro- and macro-nutrients (9, 11).

For most nutrition-related recommendations of the WHO/FAO, especially for n-3 PUFAs, sodium, fruits, and vegetables, we found a weak compliance, which is in agreement with those obtained using the DGAI. Compared to the nutritional goals of WHO/FAO, more compliers with total fat recommendations were found adhering to the DGAI due to the differences in the recommendations of these tools. However, recommendations for trans-fat, SFA, and cholesterol are the same regardless of the adherence tool used. Considering both dietary adherence measures, only about half of the total population are consumers of fibre, which could, in part, be due to low consumption of whole grain, a good source of fibre. The nutritional goals of WHO/FAO are more strict regarding sodium intake, which explains the difference in compliance when compared to the DGAI. In the case of free sugar consumption, the DGA re-commendation of ≤5% is far more strict than that of <10% recommended by the WHO/FAO. Thus, fewer individuals met the DGA recommendations than those who were complying with the recommendations of WHO/FAO.

### Limitations

Although the strength of this study lays in assessing separately each component of the DGAI 2005 in men and women, not having a standard quantitative dietary index for the Iranian populations was a major limitation. However, the modified DGAI used in this study was assessed for face validi-ty, and the results indicated the index associated with several health risks in an Iranian population (18). The DGAI and other American dietary indices have been previously used in different countries(35, 36) because dietary indices are created based on the previous knowledge of healthful diets and are applicable to different ethnic groups. However, to compensate for this limitation, we also used the dietary guidelines of WHO/FAO.

The second limitation was the use of FFQ, which, despite its common use for characterizing habitual intake, is well-recognized for its weakness in the quantification of nutrient intake (37). However, being easy to complete and analyze, the FFQs are the primary source for data collection in large epidemiologic surveys (38), being more informative of habitual intake than data on intake on a few specific days (37). The potential source of error in our use of FFQs could be from the estimation of serving sizes and lack of availability of a standardized Iranian FCT. For the validation of our FFQ, the USDA portion sizes and Iranian household measures were both used, indicating its good reliability and validity (27, 28). Regarding the use of FCTs to estimate nutrient intake quantitatively, the fundamental concept is that the nutrient content of specific foods is relatively constant, and variations may not be large enough to distort calculations (39). Also, estimates of long-term nutrient intakes obtained from FFQs reduce much of the error relating to sample-to-sample variation in compositions of nutrients. Moreover, using retrospective data collected within the framework of a cross-sectional study cannot accurately show compliance of, or adherence to, the dietary patterns to the DGAI 2005 recommendations. The final limitation was the inability to measure alcoholic beverage intakes as a DGAI component and, thus, having a maximum DGAI score of 19, rather than 20.

### Conclusions

The results of the present study revealed that the dietary patterns of most Tehranian adults were not in accordance with the recommendations of the DGAI 2005 as evident from the finding that over two-thirds of this population obtained <9.5 points, half the possible DGAI score of 19. In both sexes, the least adherence to the nutrition targets of the WHO/FAO was for n-3 PUFAs, sodium, and intake of fruits and vegetables.

Higher compliance with these dietary guidelines was positively associated with choices of healthy lifestyle and better quality of diet in Tehranian adults. The unbalanced dietary pattern of Tehranian adults is a matter of concern, requiring prompt policy revisions and dietary interventions to promote better quality of diet. We suggest further studies to determine the association between the quality of diet, assessed by the DGAI 2005 and the health outcomes using a cohort-format study.

## ACKNOWLEDGEMENTS

This work was supported by Grant No. 121 from the Research Institute for Endocrine Sciences of Shahid Beheshti University of Medical Sciences. The authors thank the participants of the Tehran Lipid and Glucose Study for their enthusiastic support and the staff of the Research Institute for Endocrine Sciences and the Tehran Lipid and Glucose Study unit. None of the authors had any personal or financial conflicts of interest.

**Appendix.** Example for 2000 kcal food guide pattern*

Food intake subscore		Score	Healthy choice subscore		Score
Fruits: recommended servings/day, 2 cups		1.0	At least 50% grains as whole grains		
	≥2 cups/day	0.5		≥50% grains serving from whole grains/day	1.0
	>0.5 and <2.0 cups/day	0.0		≥10% and <50% servings/day	0.5
	≤0.5 cup/day			<10% servings/day	0.0
Dark-green vegetables: recommended servings/week, 3 cups			Fibre intake/1,000 kcal		
	≥3 cups/week	1.0		Fibre ≥14 g	1.0
	>1.0 and <3.0 cups/week	0.5		Fibre ≥9 and <14	0.5
	≤1.0 cup/week	0.0		Fibre <9 g	0.0
Orange vegetables: recommended servings/week, 2 cups			Keep total fat >20% but <35% energy		
	≥2 cups/week	1.0		Total fat ≥20% and ≤35% energy	1.0
	>0.5 and <2.0 cups/week	0.5		Total fat <20% or >35% energy	0.0
	≤0.5 cup/week	0.0	Saturated fat less than 10% energy		
Legumes: recommended servings/week, 3 cups				<10% energy	1.0
	≥3 cups/week	1.0		≥10% and <12% energy	0.5
	>1.0 and <3.0 cups/week	0.5		≥12% energy	0.0
	≤1.0 cups/week	0.0	Limit trans-fat as much as possible		
Other vegetables: recommended servings/week, 6.5 cups				<1% energy from trans-fat	1.0
	≥6.5 cups/week	1.0		≥1% energy from trans-fat	0.0
	>2.0 and <6.5 cups/week	0.5	Cholesterol less than 300 mg/day		
	≤2.0 cups/week	0.0		<300 mg/day	1.0
Starchy vegetables: recommended servings/week, 3 cups				≥300 mg/day	0.0
	>3.5 cups/week	0.5	Percentage of serving of meat (protein) that is lean or low-fat		
	≥2.5 and ≤3.5 cups/week	1.0		≥75% protein choices lean or low-fat	0.5
	>1.0 and <2.5 cups/week			≥50% and <75% protein choices lean or low-fat	
	≤1.0 cup/week	0.5		<50% protein choices lean or low-fat	0.25
	Food intake subscore	Score		Healthy choice subscore	
Variety: based on score for fruit and vegetable items			Percentage of serving milk and milk products that are low-fat		
	≥0.5 on all vegetable and fruit items	1.0		≥75% milk choices low-fat or fat-free	0.5
	≥0.5 on 5 of 6 vegetable and fruit items	0.5		≥50% and <75% milk choices low-fat or fat-free	0.25
	≥0.5 on 4 or fewer vegetable and fruit items	0.0		<50% milk choices low-fat or fat-free	0.0
All grain: recommended servings/day, 6 oz			Sodium intake less than 2,300 mg/day		
	>6.5 oz/day	0.5		<2,300 mg sodium/day	1.0
	≥5.5 and ≤6.5 oz/day	1.0		≥2,300 mg sodium/day	0.0
	>2.0 and <5.5 oz/day	0.5		
	≤2.0 oz/day	0.0			
Meat and beans: recommended oz equivalents/day, 5.5 oz					
	>6 oz/day	0.5			
	≥5.5 and ≤6 oz/day	1.0			
	>2.0 and <5.5 oz/day	0.5			
	≤2.0 oz/day	0.0			
Milk and milk products: recommended servings/day, 3 cups					
	>3.5 cups/day	0.5			
	≥2.5 and ≤ 3.5 cups/day	1.0			
	>1.0 and <2.5 cups/day	0.5			
	≤1.0 cup/day	0.0			
Discretionary energy: % of total energy from added sugar					
	≤5% of total energy	1.0			
	>5 and <8.5% of total energy	0.5			
	≥8.5% of total energy	0.0			

*The Iranian-modified index has 19 scores since one item on alcohol consumption was not included
